# Uncovering α-synuclein and tau burden in Alzheimer’s and Lewy body diseases

**DOI:** 10.1093/braincomms/fcaf324

**Published:** 2025-09-02

**Authors:** Stephan Quintin, Giavanna Paterno, Grace M Lloyd, Stefan Prokop, Benoit I Giasson

**Affiliations:** Department of Neuroscience, College of Medicine, University of Florida, BMS J489/CTRND, 1275 Center Drive, Gainesville, FL 32610, USA; Center for Translational Research in Neurodegenerative Diseases, College of Medicine, University of Florida, Gainesville, FL 32610, USA; Department of Neuroscience, College of Medicine, University of Florida, BMS J489/CTRND, 1275 Center Drive, Gainesville, FL 32610, USA; Center for Translational Research in Neurodegenerative Diseases, College of Medicine, University of Florida, Gainesville, FL 32610, USA; Department of Neuroscience, College of Medicine, University of Florida, BMS J489/CTRND, 1275 Center Drive, Gainesville, FL 32610, USA; Center for Translational Research in Neurodegenerative Diseases, College of Medicine, University of Florida, Gainesville, FL 32610, USA; Center for Translational Research in Neurodegenerative Diseases, College of Medicine, University of Florida, Gainesville, FL 32610, USA; Department of Pathology, College of Medicine, University of Florida, Gainesville, FL 32610, USA; Norman Fixel Institute for Neurological Diseases, College of Medicine, University of Florida, Gainesville, FL 32610, USA; Department of Neuroscience, College of Medicine, University of Florida, BMS J489/CTRND, 1275 Center Drive, Gainesville, FL 32610, USA; Center for Translational Research in Neurodegenerative Diseases, College of Medicine, University of Florida, Gainesville, FL 32610, USA

**Keywords:** Alzheimer’s disease, Lewy body disease, post-translational modifications, α-synuclein, tau

## Abstract

Neurodegenerative diseases are classified based on their histopathological hallmarks alongside their clinical manifestations. Alzheimer’s disease and Lewy body disease, which includes Parkinson’s disease, are traditionally referred to as tauopathy and synucleinopathy, respectively. However, much like how Alzheimer’s disease can present with Parkinsonian features and Parkinson’s disease with cognitive impairment, there is considerable overlap in their underlying pathology. In this study, we applied antibodies specific for disease-related, post-translationally modified epitopes in α-synuclein and tau to post-mortem brain tissue from 16 Alzheimer’s disease and 9 Lewy body disease cases with a focus on the substantia nigra and the locus coeruleus. We demonstrate the presence of inclusion pathology comprised of carboxy-terminally truncated α-synuclein in the substantia nigra and locus coeruleus of Alzheimer’s disease cases that is not revealed with standard post-mortem screening. These findings suggest that α-synuclein pathology in Alzheimer’s disease can be significantly underestimated. Additionally, we observed abundant tau pathology in the substantia nigra of Alzheimer’s disease cases at levels often exceeding those seen in Lewy body disease, despite the absence of a movement disorder diagnosis in Alzheimer’s disease cases. Furthermore, the connection between regional tau pathology and associated clinical impairment may be region-dependent, where some tau pathology may be, at least temporally, innocuous.

## Teaser

Quintin *et al*. reveal abundant carboxy-truncated α-synuclein, undetected by conventional antibodies, together with tau pathology in the substantia nigra and locus coeruleus of Alzheimer’s disease brains, often greater than in Lewy body disease cases, highlighting an overlooked pathological overlap in these neurodegenerative diseases.

## Graphical Abstract

**Graphical Abstract:**
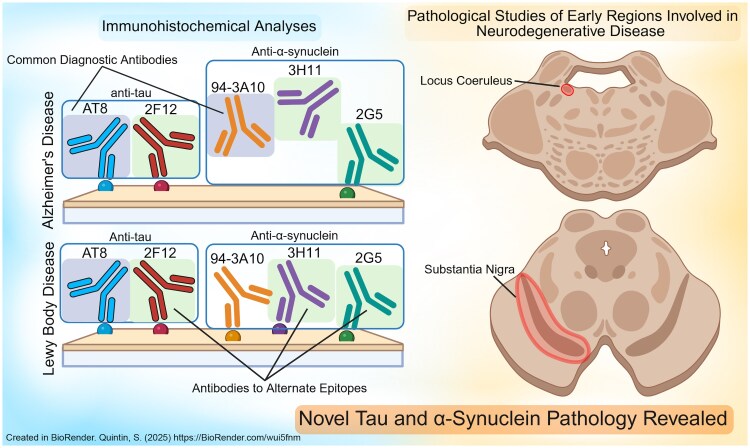

## Introduction

Alzheimer’s disease and Lewy body disease are the two most common neurodegenerative disorders, each characterized and defined by hallmark CNS pathological proteinaceous inclusions with distinct neuropathological distribution and progression. Insights from post-mortem pathological studies have shaped the current paradigm of neurodegenerative diseases, positing that aberrant protein aggregation and propagation can be central drivers of progressive physiological dysfunction and clinical decline.

Alzheimer’s disease is characterized by hyperphosphorylated tau aggregates accumulating as neurofibrillary tangles (NFTs) with concomitant amyloid-β plaques; however, in many cases, co-occurring α-synuclein pathology in the form of Lewy body (LB) pathology can be present.^1,2^ Similarly, in Parkinson’s disease and other Lewy body diseases including dementia with Lewy bodies (DLB), defined by α-synuclein inclusions, tau pathology can also be present.^3^ A recent study shed light on the potential importance of NFTs in the midbrain, where these inclusions correlated more strongly with movement disorder than LBs, challenging the current dogma of α-synuclein as a primary pathogenic mediator in PD.^4^

Tau and α-synuclein undergo extensive post-translational modifications that influence their conformation and immunohistochemical detection, complicating the accurate assessment of pathological findings but possibly revealing underlying pathogenesis.^5,6^ To this end, we apply unique tau and α-synuclein antibodies to investigate pathology in the locus coeruleus (LC) and substantia nigra (SN), two regions critically affected in Alzheimer’s disease and Lewy body disease.^7,8^ The LC, an early site of neurodegeneration and accumulation of proteinaceous inclusions in Alzheimer’s disease and Lewy body disease,^9^ has been proposed as a nidus for pathological spread through its extensive projections to the brainstem, midbrain and cortex.^10-12^ In Alzheimer’s disease and Lewy body disease, LC degeneration and tau burden are linked to cognitive decline.^8,13^ While more commonly associated with Lewy body disease, neurodegeneration of the SN is also common in Alzheimer’s disease, with almost a quarter of patients developing Parkinsonian features; however, the contribution of tau or α-synuclein pathology to this occurrence is not resolved.^14^

The present study investigated the prevalence and distribution of α-synuclein and tau pathology in the LC and SN across Alzheimer’s disease, Lewy body disease, primary age-related tauopathy (PART) and control cases. Using a unique set of antibodies, we performed immunohistochemistry (IHC) analyses for the detection of α-synuclein and tau inclusions. Our findings show an unprecedented prevalence of aggregated α-synuclein in the SN of Alzheimer’s disease cases and regional tau pathology of surprising comparative abundance between Alzheimer’s disease and Lewy body disease cases, challenging the specificity of these pathological markers to their canonically associated diseases.

## Materials and methods

### Haematoxylin and eosin staining

Formalin-fixed paraffin-embedded human brain tissue was obtained from the University of Florida Neuromedicine Brain and Tissue Bank (UF HBTB) (Supplementary Table 1). Sections were deparaffinized in xylenes and rehydrated in a descending series of ethanol solutions (100, 100, 90, 70%) followed by water. Slides were incubated in haematoxylin and then washed in tap water. Slides were then incubated in 90% ethanol for 1 min, followed by Eosin Y-solution 0.5% alcoholic (Sigma-Aldrich, St. Louis, MO, USA) with 0.2% glacial acetic acid, followed by washes in several exchanges of 100% ethanol, xylenes and coverslipped.

### Immunohistochemistry

For IHC, paraffin-embedded human brain sections were processed following previously published protocols for each antibody, as outlined in Supplementary Table 2.

### Independent clinicopathologic diagnosis of study cases

Post-mortem diagnoses of Lewy body disease and Alzheimer’s disease neuropathological change and other changes were made according to current guidelines and criteria proposed by the National Institute of Aging-Alzheimer’s Association,^15^ the Dementia with Lewy Bodies Consortium.^16^

### Analysis of immunohistochemical staining

For analysis of staining and capturing representative images, slides were scanned on an Aperio AT2 scanner (Leica Biosystems) at 40 × magnification. Slide images were analysed using the QuPath platform (Version 0.5.1), running Windows 10. Regions of interest (ROI), including the SN and the LC, were identified on haematoxylin and Eosin (H&E)-stained images and annotated on IHC-stained images accordingly. A manual analysis of staining was done for each case and ROI to determine whether pathology was present for which an assignment of positive or negative was given. Positivity in an ROI was defined by the presence of one or more inclusions, including globular or thread-like morphological features (Supplementary Fig. 1). Following the exclusion of tissue and stain artefacts, the ‘Pixel Classifier’ tool was used to detect and quantify stained pathology. The percentage of annotated ROI covered by pathological staining was determined as (positive pixel count / pixel count of ROI) × 100. Thresholds for positive staining were created under the following general settings: ‘resolution’, full (0.25 um/px); ‘channel’, DAB; ‘prefilter’, Gaussian. Thresholds for DAB positivity and staining vectors for DAB were adjusted on an image-by-image basis to ensure no false positive staining (Supplementary Fig. 1).

### Statistical analysis

Statistical analysis was performed using GraphPad Prism 10 software (GraphPad Software). Spearman’s ranked correlation was performed for all correlative analyses, and the Kruskal–Wallis test was used for group comparisons of quantified staining.

## Results

### Clinical and neuropathologic characteristics of study groups

For the comparative assessment of pathology, we included 16 Alzheimer’s disease cases, 9 Lewy body disease cases, 6 PART cases and 8 control cases with prior clinical and neuropathological diagnoses (Supplementary Table 1). Due to limited tissue availability, staining for the SN was not available for two Lewy body disease cases, and one control case, while the LC was unavailable for one Alzheimer’s disease case.

As expected, concomitant brain neuropathologies were present in some Alzheimer’s disease, Lewy body disease and PART cases.^2^ The secondary neuropathological profiles observed in this study align with previous findings.^2^ However, none of the Alzheimer’s disease cases selected for the current study exhibited previously identified Lewy body pathology.

### Prevalence of α-synuclein and tau pathology in the locus coeruleus and substantia nigra

Haematoxylin and eosin (H&E) staining was performed on all cases to identify regions of interest, which were annotated accordingly (Supplementary Fig. 1). IHC was conducted using α-synuclein antibodies: 2G5 (specific to carboxy-terminal truncation at residue 103), 94-3A10 (targeting C-terminal residues 130–140) and 3H11 (targeting residues 43–62) (Supplementary Table 2). Tau IHC was performed using the AT8 antibody for tau phosphorylated at Ser202 and Thr205 (p-tau) and the conformational tau antibody 2F12 (Supplementary Table 2). Importantly, antibody AT8 has been commonly used in spatial and temporal studies of human tau pathology.^17,18^ Antibodies 2G5, 3H11 and 2F12 target less conventional epitopes that can reveal novel pathological features.

**Figure 1:**
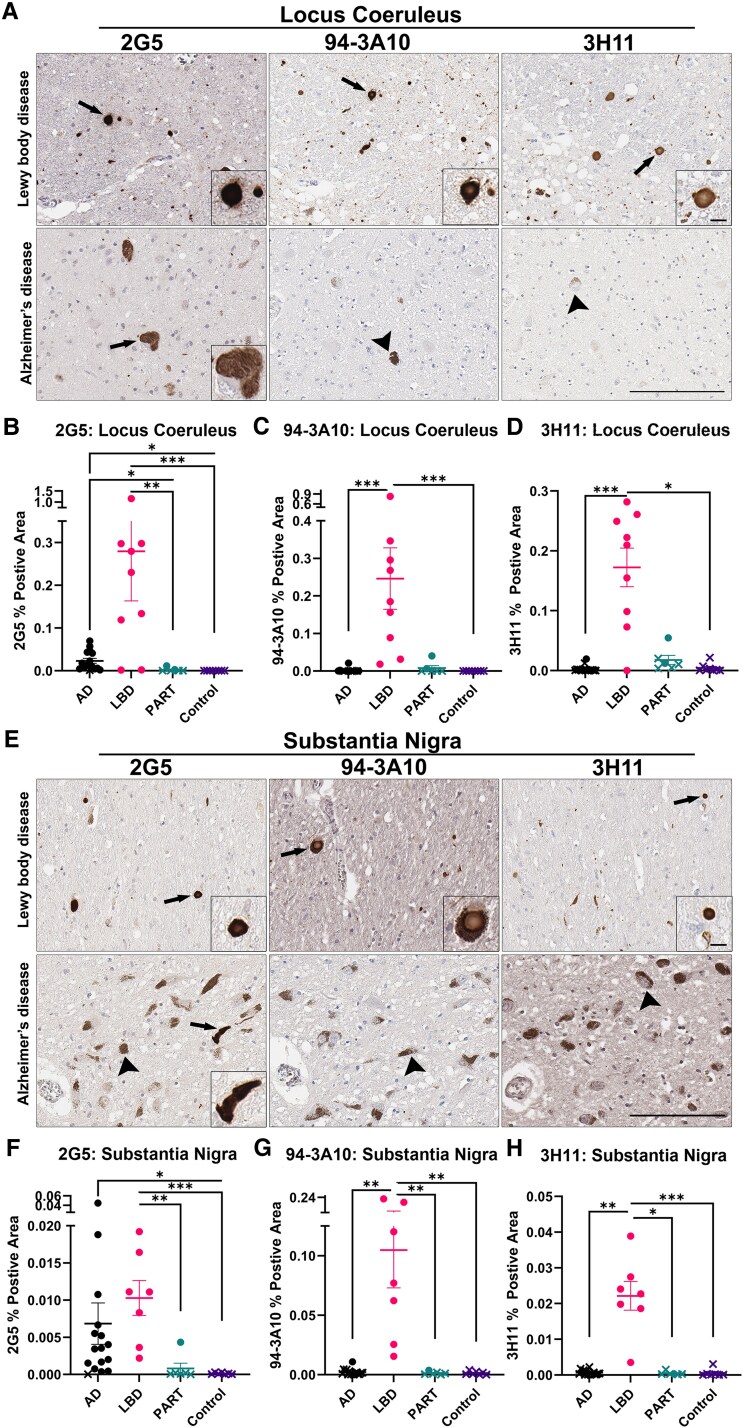
**α-Synuclein pathological inclusions in the locus coeruleus and substantia nigra of Alzheimer’s disease and Lewy body disease cases.** (**A**) Representative images of 2G5, 94-3A10 and 3H11 immunostaining in the LC of Lewy body disease and Alzheimer’s disease cases. (**B**) Quantification of 2G5 inclusion pathology in the LC of Alzheimer’s disease (*N* = 15), Lewy body disease (*N* = 9), PART (*N* = 6) and control cases (*N* = 8). (**C**) Quantification of 94-3A10 inclusion pathology in the LC of Alzheimer’s disease, Lewy body disease, PART and control cases. (**D**) Quantification of 3H11 inclusion pathology in the LC of Alzheimer’s disease, Lewy body disease, PART and control cases. (**E**) Representative images of 2G5, 94-3A10 and 3H11 immunostaining in the SN of Lewy body disease and Alzheimer’s disease cases. (**F**) Quantification of 2G5 inclusion pathology in the SN of Alzheimer’s disease (*N* = 16), Lewy body disease (*N* = 7), PART (*N* = 6) and control cases (*N* = 7). (**G**) Quantification of 94-3A10 inclusion pathology in the SN of Alzheimer’s disease, Lewy body disease, PART and control cases. (**H**) Quantification of 3H11 inclusion pathology in the SN of Alzheimer’s disease, Lewy body disease, PART and control cases. Arrows indicate inclusions. Arrowheads depict neuromelanin-laden neurons. Large image scale bar = 100 µm, inset scale bar = 10 µm. On graphed quantifications, data point shape indicates whether a case is positive or negative for a said pathology as determined during manual examination, with circles indicating positive cases and ×’s indicating negative cases. Kruskal–Wallis test was used for group comparisons. Error bars = SEM. ^∗^*P* ≤ 0.05; ^∗∗^*P* ≤ 0.01, ^∗∗∗^*P* ≤ 0.001.

Pathology was classified as positive or negative based on antibody staining (Table 1, Supplementary Table 3, Supplementary Table 4). All Lewy body disease cases presented with α-synuclein inclusions positive for 2G5, 3H11 and 94-3A10 in the LC and SN (Fig. 1; Table 1, Supplementary Table 3). Unexpectedly, all but one Alzheimer’s disease case was positive for 2G5 pathology in the LC and the SN (Fig. 1, Table 1, Supplementary Table 3). However, only one Alzheimer’s disease case (AD-16) displayed positivity for all three α-synuclein antibodies in both regions.

**Table 1:** Fraction of cases with positive staining in ROI per disease group

	2G5 (α-synuclein)		94-3A10 (α-synuclein)		3H11 (α-synuclein)		AT8 (tau)		2F12 (tau)	
	LC	SN	LC	SN	LC	SN	LC	SN	LC	SN
**Alzheimer’s disease**	14/15	15/16	1/15	1/16	1/15	1/16	15/15	16/16	15/15	16/16
**Lewy body disease**	9/9	7/7	9/9	7/7	9/9	7/7	9/9	7/7	9/9	6/7
**PART**	2/6	1/6	2/6	1/6	2/6	1/6	5/6	1/6	5/6	3/6
**Control**	0/8	0/7	0/8	0/7	0/8	0/7	6/8	3/7	6/8	1/7

LC, locus coeruleus; SN, substantia nigra; PART, primary age-related tauopathy.

Two PART cases (PART-1 and PART-2) exhibited α-synuclein pathology in the LC and SN (Table 1, Supplementary Fig. 4, Supplementary Table 3). These cases showed positivity for all three α-synuclein antibodies in the LC, but their SN staining patterns differed: PART-1 was positive for 2G5 but negative for 94-3A10 and 3H11, while PART-2 was positive for 94-3A10 and 3H11 but negative for 2G5 (Supplementary Table 3). In contrast, no control cases showed α-synuclein positivity in either brain region (Fig. 1, Supplementary Table 3).

**Figure 2:**
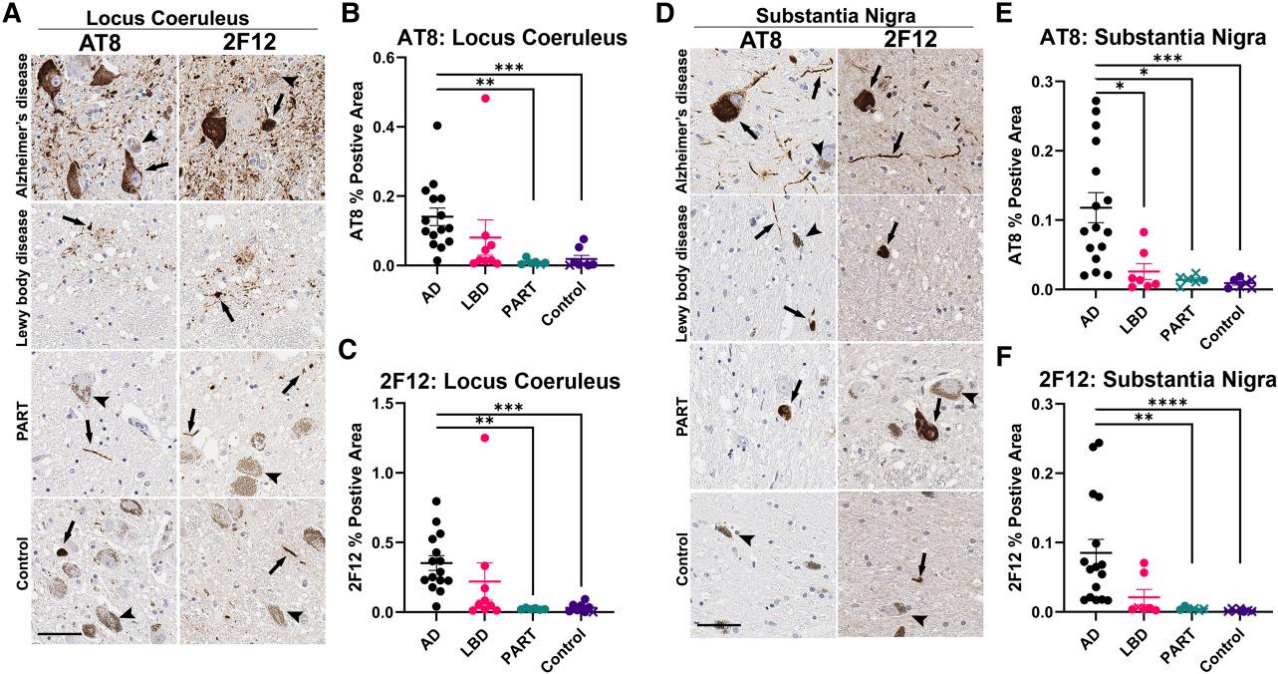
**Tau pathology immunoreactivity in the locus coeruleus and substantia nigra of Alzheimer’s disease, Lewy body disease, primary age-related tauopathy and control cases.** (**A**) Representative images of AT8 and 2F12 staining in the LC of Alzheimer’s disease, Lewy body disease, PART and control cases. (**B**) Quantification of AT8 inclusion pathology in the LC of Alzheimer’s disease (*N* = 15), Lewy body disease (*N* = 9), PART (*N* = 6) and control cases (*N* = 8). (**C**) Quantification of 2F12 inclusion pathology in the LC of Alzheimer’s disease, Lewy body disease, PART and control cases. (**D**) Representative images of AT8 and 2F12 staining in the SN of Alzheimer’s disease, Lewy body disease, PART and control cases. (**E**) Quantification of AT8 inclusion pathology in the SN of Alzheimer’s disease (*N* = 16), Lewy body disease (*N* = 7), PART (*N* = 6) and control cases (*N* = 7). (**F**) Quantification of 2F12 inclusion pathology in the SN of Alzheimer’s disease, Lewy body disease, PART and control cases. Arrows indicate inclusions, and arrowheads indicate neuromelanin-rich neurons. Scale bar = 100 µm. On graphed quantifications, data point shape indicates whether a case is positive or negative for a said pathology as determined during manual examination, with circles indicating positive cases and ×’s indicated negative cases. Kruskal–Wallis test was used for group comparisons. Error bars = SEM. ^∗^*P* ≤ 0.05; ^∗∗^*P* ≤ 0.01, ^∗∗∗^*P* ≤ 0.001, ^∗∗∗^*P* ≤ 0.0001.

Tau pathology (AT8 and 2F12 positivity) was observed across all Alzheimer’s disease cases in the LC and SN regions (Fig. 2, Supplementary Table 4). Similarly, all Lewy body disease cases exhibited AT8 and 2F12 positivity, except for LBD-1, which lacked positive 2F12 staining in the SN (Supplementary Table 4). Among PART and control cases, AT8- and 2F12-positive staining in the LC was more frequent than in the SN (Fig. 2, Supplementary Table 4).

### Quantitative analysis of α-synuclein staining in the locus coeruleus and substantia nigra

Qupath tissue analysis software was used to quantify and highlight pathology within an annotated region of interest (Supplementary Fig. 1). Percent positivity was calculated as the positive pixel count divided by the pixel count of the region of interest multiplied by 100. To ensure accuracy, positive pixel thresholds were set for each case to minimize false positive detection (Supplementary Fig. 2).

Lewy body disease cases exhibited diverse Lewy pathology with characteristic globular and thread-like α-synuclein inclusions across the LC and SN (Fig. 1A and E). 2G5-positive inclusions in Alzheimer’s disease cases were morphologically similar to those in Lewy body disease cases (Fig. 1A and E, Supplementary Fig. 3). As expected, most Lewy body disease cases had higher 2G5, 94-3A10 and 3H11 positivity in the LC than that of Alzheimer’s disease cases (Fig. 1B–D). PART cases also had relatively lower pathology in the LC than most Lewy body disease cases. While this observation remained true for 94-3A10 and 3H11 staining in the SN of positive Alzheimer’s disease and PART cases (Fig. 1G and H), 2G5 per cent positivity in the SN of Alzheimer’s disease cases was in a similar range to that of Lewy body disease cases, with one Alzheimer’s disease case exceeding that of any Lewy body disease case (Fig. 1F).

### Quantitative assessment of tau staining in the locus coeruleus and substantia nigra

In the present study, quantitative analysis of AT8 and 2F12 in Alzheimer’s disease and Lewy body disease cases revealed abundant positivity in both regions, with Alzheimer’s disease cases often surpassing that of Lewy body disease cases (Fig. 2). Surprisingly, some PART cases exhibited a lower tau burden than control cases, with inclusions so sparse that quantified positivity rarely exceeded background levels of negative cases (Fig. 2B, C, E, F).

### Correlation analysis of α-synuclein and tau staining

**Figure 3:**
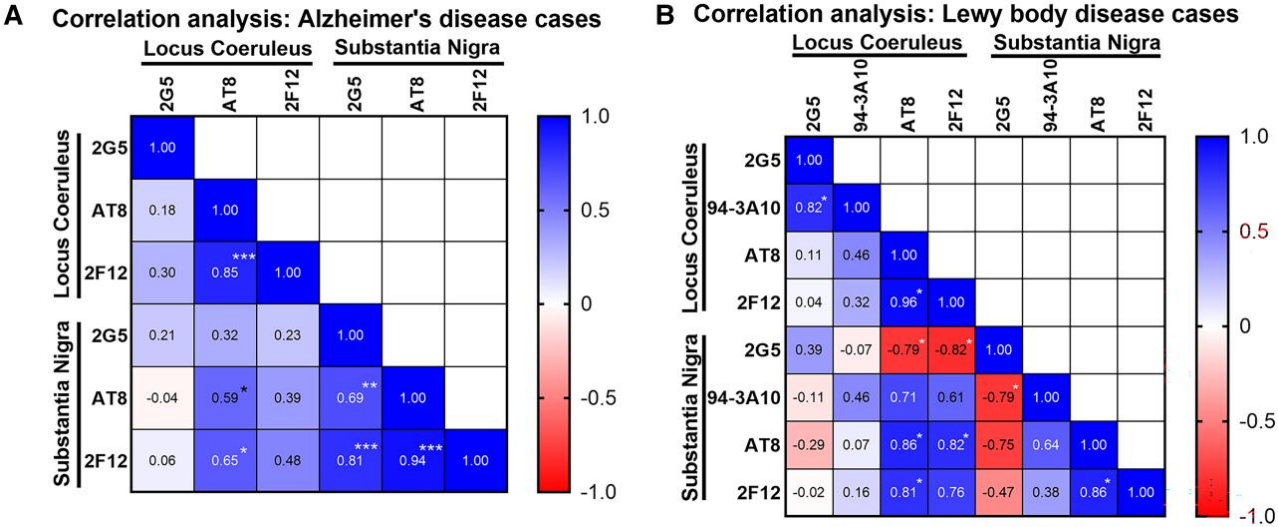
**Correlation and relative intensity of α-synuclein and tau pathologies in the LC and SN of Lewy body disease and Alzheimer’s disease cases.** (**A**) Correlation matrix of Spearman’s rank-based correlation analysis of 2G5, AT8 and 2F12 inclusion pathology in the LC (*n* = 15) and SN (*n* = 16) of Alzheimer’s disease cases with correlation coefficients displayed. (**B**) Correlogram of Spearman’s rank-based correlation analysis of 2G5, 94-3A10, AT8 and 2F12 inclusion pathology in the LC (*n* = 9) and SN (*n* = 7) of Lewy body disease cases with correlation coefficients displayed. Correlation analysis performed using quantified pathology (area covered) as detected by respective antibodies on stained sections. *P≤ 0.05; **P ≤ 0.01; ***P ≤ 0.001.

Spearman’s ranked correlation analysis of 2G5, AT8 and 2F12 staining in the LC and SN of Alzheimer’s disease cases revealed significant interregional correlations for AT8 staining, with LC AT8 positively correlating with SN AT8 and 2F12 (Fig. 3A). Within regions, the strongest correlation was in the SN between 2G5, AT8 and 2F12. In the LC, only AT8 and 2F12 were significantly correlated (Fig. 3A).

Among Lewy body disease cases, AT8 staining in the LC and SN was positively correlated, as was LC 2F12 with SN AT8. However, LC AT8 and 2F12 staining was inversely correlated with SN 2G5 (Fig. 3B). 2G5 and 94-3A10 staining were positively correlated in the LC but inversely correlated in the SN. Unlike that of Alzheimer’s disease cases, 2G5 staining was inversely correlated with AT8 and 2F12 staining in the SN of Lewy body disease cases (Fig. 3B).

## Discussion

The present report brings new data that challenge the prominent dogmatic views of tau and α-synuclein pathology and their isolated roles in neurodegeneration. We assessed the LC, an early region to accumulate tau and α-synuclein inclusion pathology in Alzheimer’s disease and Lewy body disease, respectively.^9,10^ We also assess the SN, which is primarily associated with neurodegeneration in Lewy body disease. Our data demonstrates the prominence of pathological inclusions comprised of carboxy-terminally cleaved α-synuclein in SN and LC of Alzheimer’s disease cases. The presence of LBs in Alzheimer’s disease cases has been associated with more severe cognitive decline than that of ‘pure’ Alzheimer’s disease cases; however, LBs in primary Alzheimer’s disease are largely restricted to the amygdala rather than the SN or LC.^19,20^ Findings here show that LB may be more ubiquitous in Alzheimer’s disease, but the clinical implications of these highly cleaved α-synuclein inclusions are unclear. Nevertheless, a high prevalence and the broader association between α-synuclein pathology and neurodegenerative disease bring into question whether it has a more prominent role in Alzheimer’s disease.^21^

The extent to which α-synuclein pathology in Lewy body disease corresponds to neurodegeneration remains a contentious presumption, with some studies implicating tau rather than α-synuclein as a disease correlate.^22^ The present study shows that SN and LC tau pathology are less specific to neurodegenerative disease than α-synuclein pathology. We show that Alzheimer’s disease cases, without clinical histories of parkinsonism, have abundant tau and carboxy-truncated α-synuclein pathology in the LC and SN, with tau pathology often superseding that of Lewy body disease patients. In addition to newly revealed α-synuclein pathology, these data bring into question the pathogenic implications of tau pathology in these regions. One might expect that abundant tau pathology in the SN of Alzheimer’s disease cases would correspond to Parkinsonism, but our additional findings of tau pathology in PART and control cases also support the notion that some tau inclusions could be, at least temporarily, benign—a concept that was previously proposed.^23,24^ Others have also recently reported tau pathology in the SN of Alzheimer’s disease patients without overt motor disorders and conversely movement disorder in Alzheimer’s disease patients without α-synuclein pathology.^25^ Further investigation is needed to clarify the relationship of SN tau pathology to neurodegeneration and clinical outcomes in each disease context.

Our findings further demonstrate the histopathological overlap between Alzheimer’s disease and Lewy body disease and highlight the potential insufficiency of the terms tauopathy and synucleinopathies as categorical descriptors of the most prominent neurodegenerative diseases. However, a broader survey of carboxy-terminally truncated α-synuclein pathology is needed to make definitive statements about the prevalence of such pathology. The present study is limited in scope regarding the number of cases included and the degree of sampling from each region of interest. While post-mortem IHC remains the gold standard for detecting these pathologies, it provides only a static snapshot of pathology, limiting insights into the temporal dynamics between co-occurring pathologies, neurodegeneration and clinical symptoms. Furthermore, the fidelity of histological findings depends on the sensitivity and specificity of the tools employed, introducing potential tool-dependent biases in studies attempting to survey the prevalence of pathologies. By employing PTM-specific antibodies, this study provides compelling evidence for an underappreciated prevalence of some forms of α-synuclein pathology in Alzheimer’s disease and highlights the need for a reassessment of SN tau pathology in neurodegenerative diseases.

## Supplementary Material

fcaf324_Supplementary_Data

## Data Availability

The datasets used and analysed from the current study are available from the corresponding author upon reasonable request.
