# Supplementary material for: Uncovering α-synuclein and tau burden in Alzheimer’s and Lewy body diseases
Source: Brain Commun. 2025 Sep 2;7(5):fcaf324. doi: 10.1093/braincomms/fcaf324 (PMC12507090; doi:10.1093/braincomms/fcaf324)
Supplement: fcaf324_Supplementary_Data [file fcaf324_supplementary_data.pdf]

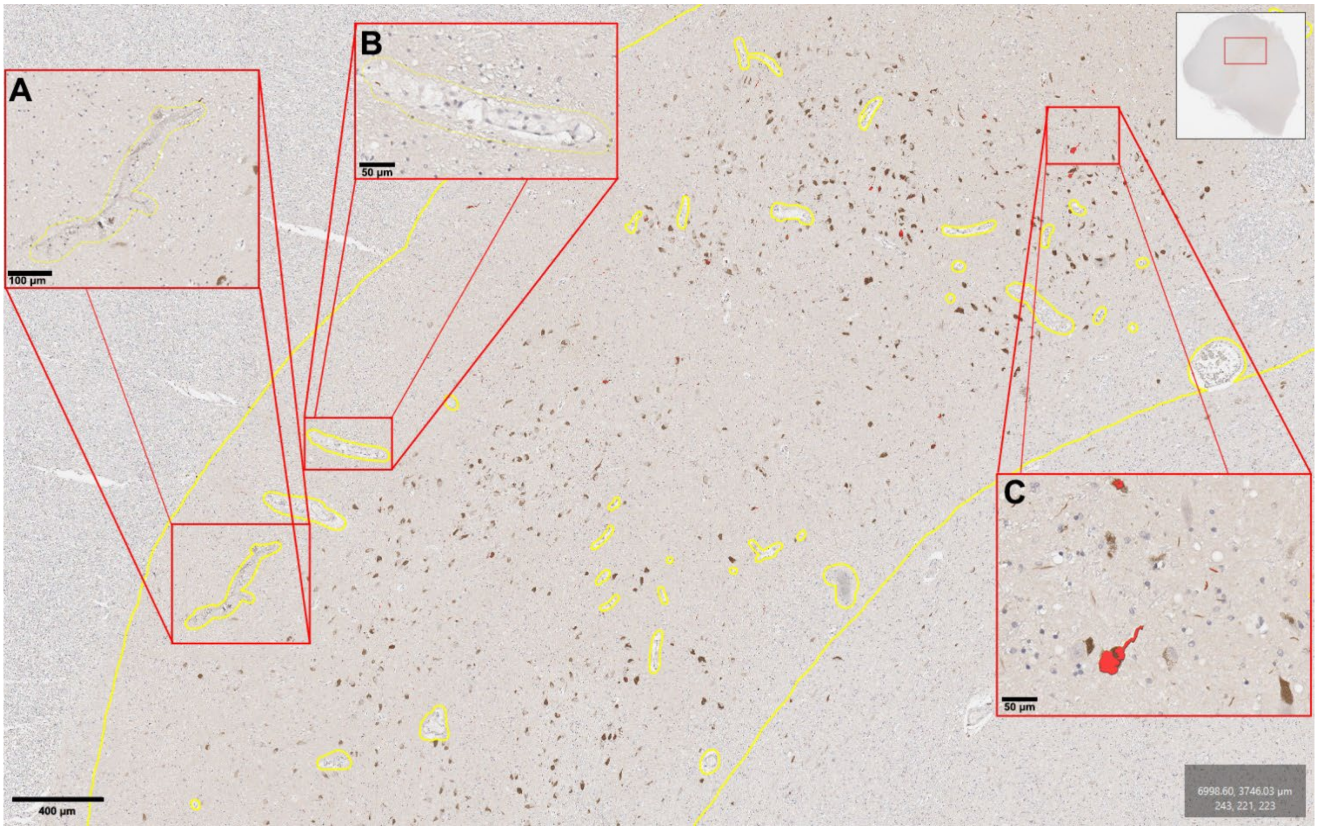

Supplemental Figure 1: Representative image of regional annotation with excluded artifacts and neuromelanin-laden neurons. Inset **A** shows a tissue artifact excluded from analysis as outlined in yellow. Inset **B** shows a vessel that is excluded from analysis as outlined in yellow. Inset **C** shows a representative inclusion found by immunohistochemical staining with antibody 2G5, which is highlighted in red by positive pixel detection in Qupath software.

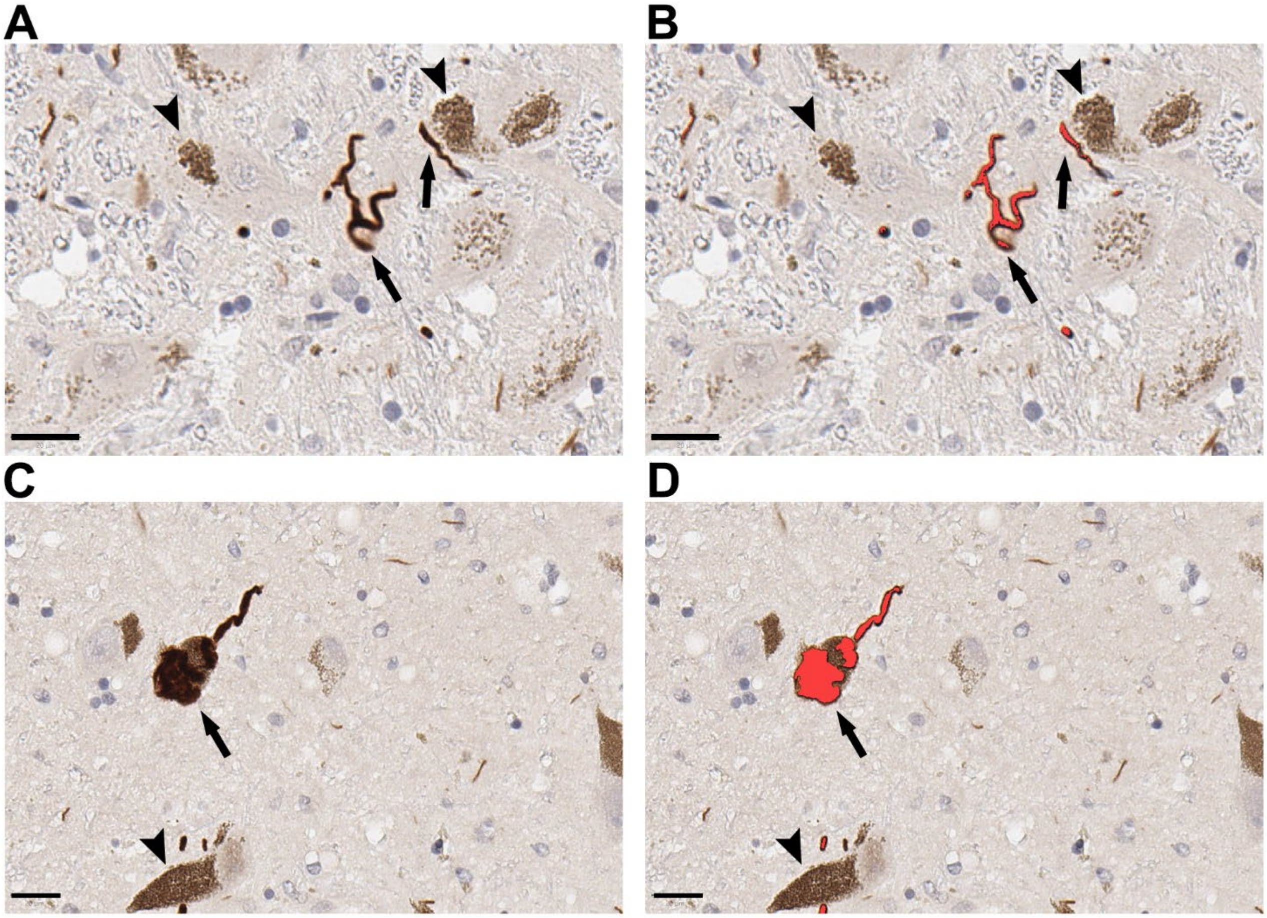

Supplemental Figure 2: Representative images of positive pixel detection used for quantitative analysis of immunohistochemical staining. 2G5-positive thread-shaped inclusions **(arrows)** amid neuromelanin-neurons **(arrowheads)** shown in **A** are selectively detected as highlighted by the positive pixel detection in **B**. Globular inclusions **(arrows)** within neuromelanin-laden neurons **(arrowheads)** in **C** are selectively detected as highlighted in red **D**. Scale bars = 20 µm.

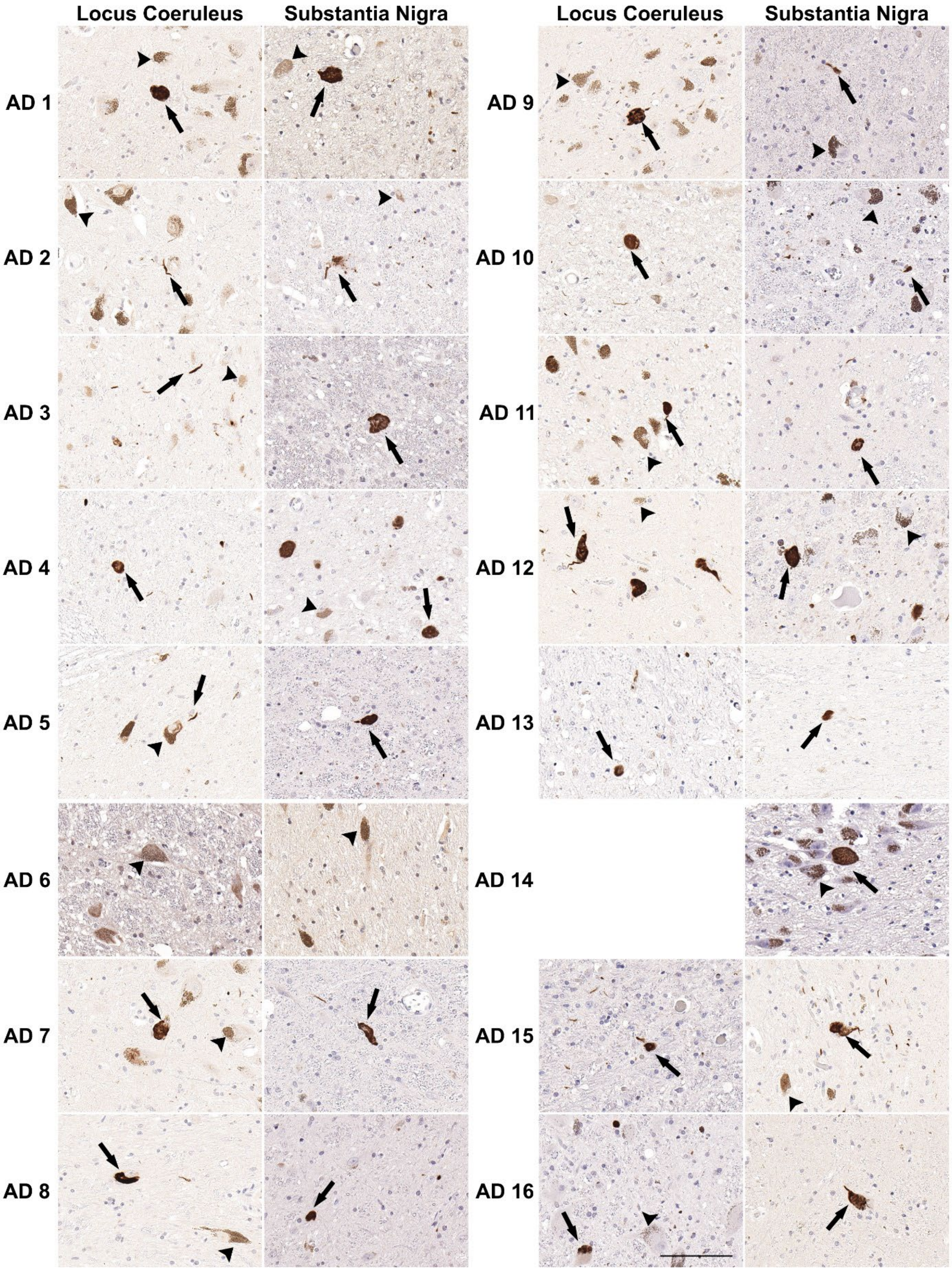

Supplemental Figure 3: Representative images of 2G5 positive staining from Alzheimer's disease cases. Case number indicated to the left of representative images from the substantia nigra and locus coeruleus of each case. Arrows indicate 2G5 positive inclusions. Arrowheads indicate neuromelanin-laden neurons. Absence of representative image is due to tissue being unavailable for corresponding case and region. Scale bar = 100 μm.

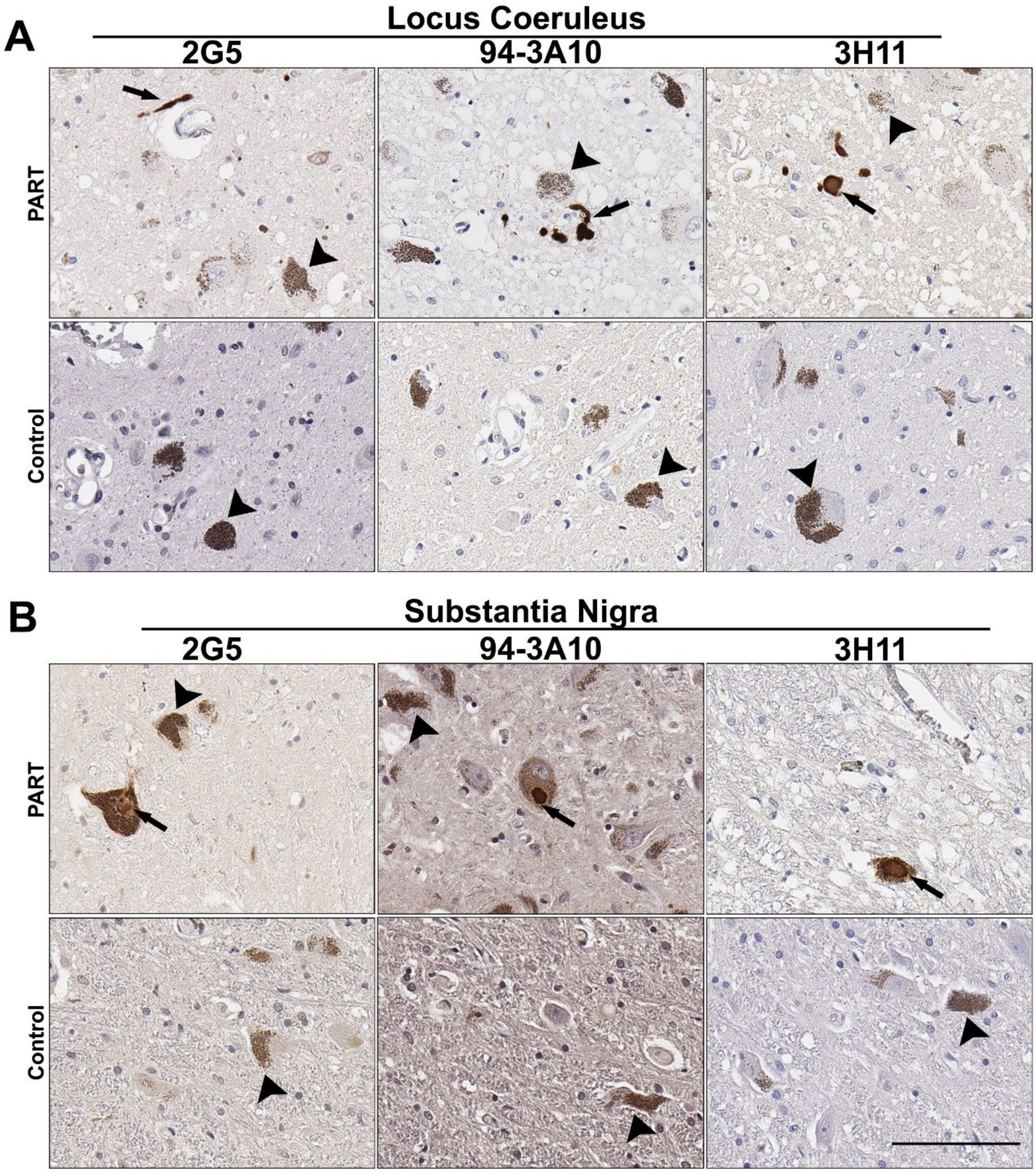

Supplemental Figure 4: (A) Representative images of 2G5, 94-3A10, and 3H11 staining in the locus coeruleus of PART and control cases. **(B)** Representative images of 2G5, 94-3A10, and 3H11 staining in the substantia nigra of PART and control cases. Arrows indicate inclusions. Arrowhead indicates neuromelanin-laden neurons. Scale bar = 100µm.

Case	Clinical-pathological diagnosis	Primary neuropathological diagnosis	Secondary neuropathological diagnosis	Thal	Braak	Cerad	Sex	Age	Region(s) investigated	Braak PD	SN neuronal loss
AD-1	AD	AD high	CAA widespread, moderate	5	V	frequent	m	83	LC, SN	N/A	1
AD-2	AD	AD high	CAA focal, moderate	4	V	frequent	m	71	LC, SN	N/A	1
AD-3	AD	AD high	CAA focal, mild	5	V	frequent	f	85	LC, SN	N/A	1
AD-4	AD	AD high	CAA focal, mild	5	V	frequent	f	83	LC, SN	N/A	0
AD-5	AD	AD high	CAA widespread, mild to moderate	5	V	frequent	f	83	LC, SN	N/A	1
AD-6	AD	AD high		4	V	frequent	f	85	LC, SN	N/A	0
AD-7	AD	AD high	CAA focal, moderate	5	VI	frequent	m	63	LC, SN	N/A	0
AD-8	AD	AD high	CAA widespread, moderate	5	V	frequent	f	78	LC, SN	N/A	1
AD-9	AD	AD high	CAA widespread, mild to moderate	5	V	frequent	f	97	LC, SN	N/A	0
AD-10	AD	AD high	CAA focal, moderate	5	V	frequent	m	95	LC, SN	N/A	1
AD-11	AD	AD high	CAA focal, mild	5	V	frequent	m	78	LC, SN	N/A	0
AD-12	AD	AD high	CAA widespread, moderate	5	VI	frequent	m	83	LC, SN	N/A	0
AD-13	AD	AD high		4	V	frequent	f	93	LC, SN	N/A	1
AD-14	AD	AD high	CAA widespread, moderate	5	VI	frequent	m	72	SN	N/A	1
AD-15	AD	AD high	CAA focal, moderate	5	VI	frequent	f	63	LC, SN	N/A	1
AD-16	AD	AD high	CAA widespread, moderate	4	VI	frequent	m	75	LC, SN	N/A	1
LBD-1	DLB	LBD diffuse neocortical	PART, Braak II	0	II	none	f	87	LC, SN	5	2
LBD-2	DLB	LBD diffuse neocortical	PART, Braak II	0	II	none	m	62	LC, SN	5	3
LBD-3	DLB	LBD diffuse neocortical	AD int.	3	III	moderate	m	83	LC, SN	5	3
LBD-4	DLB	LBD diffuse neocortical	AD low	3	I	none	m	69	LC, SN	5	3
LBD-5	DLB	LBD diffuse neocortical	AD low	3	II	sparse	m	79	LC, SN	5	3
LBD-6	DLB	LBD diffuse neocortical	AD int.	4	III	sparse	m	67	LC, SN	5	1
LBD-7	DLB	LBD diffuse neocortical	AD high	5	V	frequent	f	68	LC, SN	5	3
LBD-8	DLB	LBD diffuse neocortical	AD int.	3	III	sparse	m	77	LC	5	not available
LBD-9	DLB	LBD diffuse neocortical	AD low	3	II	none	m	80	LC	5	not available
PART-1	cognitive issues	PART, definite, Braak II	CAA focal, mild to moderate	0	II	none	m	75	LC, SN	N/A	1
PART-2	PART	PART, definite, Braak II		0	II	none	f	72	LC, SN	N/A	1
PART-3	PART	PART, Braak I	CAA widespread, mild	0	I	none	m	71	LC, SN	N/A	1
PART-4	PART	PART, definite, Braak II	subacute microinfarct corpus callosum	0	II	none	f	90	LC, SN	N/A	1
PART-5	PART	PART, definite, Braak I		0	I	none	f	78	LC, SN	N/A	0
PART-6	PART	PART, definite, Braak I		0	I	none	m	74	LC, SN	N/A	1
Control-1	suspected CTE	No significant pathological findings		0	0	none	m	53	LC, SN	N/A	0

Control-2	Metastatic cancer	No significant pathological findings	Rare Abeta plaques in neocortex	1	0	none	m	46	LC, SN	N/A	1
Control-3	Metastatic melanoma	Metastatic melanoma	AD low	2	0	none	m	61	LC, SN	N/A	1
Control-4	Control	No significant pathological findings		0	0	none	f	55	LC, SN	N/A	1
Control-5	CTSB mutation	No significant pathological findings		0	0	none	m	51	LC, SN	N/A	1
Control-6	Control	Atherosclerosis (moderate)		0	0	none	m	71	LC	N/A	1
Control-7	Control	Acute microinfarcts		0	0	none	m	72	LC, SN	N/A	0
Control-8	GBM	Glioblastoma, WHO grade IV left frontal		0	0	none	m	60	LC, SN	N/A	1

Supplemental Table 1. Listed are the clinical and pathological diagnoses, the sex, age at death, Thal phase, Braak stage, and CERAD ratings. AD Alzheimer's disease, CAA cerebral amyloid angiopathy, CERAD Consortium to Establish a Registry for Alzheimer's disease, CTE chronic traumatic encephalopathy, DLB dementia with Lewy bodies, LC locus coeruleus. PART primary age-related tauopathy, SN substantia nigra. * CTSB mutation, Braak PD: 1-5 or N/A (not applicable-no pathology), Substantia nigra neuronal loss: 0-negligible, 1-mild, 2-moderate, 3-severe

	Antibody	Host	Epitope (residues)	Dilution	Antigen retrieval for IHC	References
**α-synuclein**	3H11	Mouse	43-62	1:1000	DAKO Target Retrieval Solution, heat bath /70% [illegible]	1
	94-3A10	Mouse	130-140	1:1000	Water, heat bath /70% formic acid	1
	2G5	Mouse	103 carboxyl end specific	1:1000	DAKO Target Retrieval Solution, heat bath /70% formic acid	2
**Tau**	AT8	Mouse	pS202 & pT205	1:1000	None	3
	2F12	Mouse	218-222 (conformation-dependent)	1:1000	DAKO Target Retrieval Solution, heat bath	4

Supplemental Table 2. Antibodies used in this study.

1. Dhillon JKS, Riffe C, Moore BD, et al. A novel panel of α-synuclein antibodies reveal distinctive staining profiles in synucleinopathies. *PLoS One*. 2017;12(9):e0184731. doi:10.1371/journal.pone.0184731

2. Hass EW, Sorrentino ZA, Xia Y, et al. Disease-, region- and cell type specific diversity of α-synuclein carboxy terminal truncations in synucleinopathies. *Acta Neuropathol Commun*. 2021;9(1):1-18. doi:10.1186/s40478-021-01242-2

3. Mercken M, Vandermeeren M, Lübke U, et al. Monoclonal antibodies with selective specificity for Alzheimer Tau are directed against phosphatase-sensitive epitopes. *Acta Neuropathol*. 1992;84(3):265-272. doi:10.1007/BF00227819

4. Paterno G, Torrellas J, Bell BM, et al. Novel Conformation-Dependent Tau Antibodies Are Modulated by Adjacent Phosphorylation Sites. *Int J Mol Sci*. 2023;24(18). doi:10.3390/ijms241813676

	2G5		94-3A10		3H11	
**Case**	Locus Coeruleus	Substantia Nigra	Locus Coeruleus	Substantia Nigra	Locus Coeruleus	Substantia Nigra
AD-1	Positive	Positive	Negative	Negative	Negative	Negative
AD-2	Positive	Positive	Negative	Negative	Negative	Negative
AD-3	Positive	Positive	Negative	Negative	Negative	Negative
AD-4	Positive	Positive	Negative	Negative	Negative	Negative
AD-5	Positive	Positive	Negative	Negative	Negative	Negative
AD-6	Negative	Negative	Negative	Negative	Negative	Negative
AD-7	Positive	Positive	Negative	Negative	Negative	Negative
AD-8	Positive	Positive	Negative	Negative	Negative	Negative
AD-9	Positive	Positive	Negative	Negative	Negative	Negative
AD-10	Positive	Positive	Negative	Negative	Negative	Negative
AD-11	Positive	Positive	Negative	Negative	Negative	Negative
AD-12	Positive	Positive	Negative	Negative	Negative	Negative
AD-13	Positive	Positive	Negative	Negative	Negative	Negative
AD-14	N/A	Positive	N/A	Negative	N/A	Negative
AD-15	Positive	Positive	Negative	Negative	Negative	Negative
AD-16	Positive	Positive	Positive	Positive	Positive	Positive
LBD-1	Positive	Positive	Positive	Positive	Positive	Positive
LBD-2	Positive	Positive	Positive	Positive	Positive	Positive
LBD-3	Positive	Positive	Positive	Positive	Positive	Positive
LBD-4	Positive	Positive	Positive	Positive	Positive	Positive
LBD-5	Positive	Positive	Positive	Positive	Positive	Positive
LBD-6	Positive	Positive	Positive	Positive	Positive	Positive
LBD-7	Positive	Positive	Positive	Positive	Positive	Positive
LBD-8	Positive	N/A	Positive	N/A	Positive	N/A
LBD-9	Positive	N/A	Positive	N/A	Positive	N/A
PART-1	Positive	Positive	Positive	Negative	Positive	Negative
PART-2	Positive	Negative	Positive	Positive	Positive	Positive
PART-3	Negative	Negative	Negative	Negative	Negative	Negative
PART-4	Negative	Negative	Negative	Negative	Negative	Negative
PART-5	Negative	Negative	Negative	Negative	Negative	Negative
PART-6	Negative	Negative	Negative	Negative	Negative	Negative
Control-1	Negative	Negative	Negative	Negative	Negative	Negative
Control-2	Negative	Negative	Negative	Negative	Negative	Negative
Control-3	Negative	Negative	Negative	Negative	Negative	Negative
Control-4	Negative	Negative	Negative	Negative	Negative	Negative
Control-5	Negative	Negative	Negative	Negative	Negative	Negative
Control-6	Negative	N/A	Negative	N/A	Negative	N/A
Control-7	Negative	Negative	Negative	Negative	Negative	Negative
Control-8	Negative	Negative	Negative	Negative	Negative	Negative

Supplemental Table 3. Case-by-case manual categorization based on the presence (positive) or absence (negative) of α-synuclein pathology. N/A indicates a region of interest is not available.

	AT8		2F12	
Case	Locus Coeruleus	Substantia Nigra	Locus Coeruleus	Substantia Nigra
AD-1	Positive	Positive	Positive	Positive
AD-2	Positive	Positive	Positive	Positive
AD-3	Positive	Positive	Positive	Positive
AD-4	Positive	Positive	Positive	Positive
AD-5	Positive	Positive	Positive	Positive
AD-6	Positive	Positive	Positive	Positive
AD-7	Positive	Positive	Positive	Positive
AD-8	Positive	Positive	Positive	Positive
AD-9	Positive	Positive	Positive	Positive
AD-10	Positive	Positive	Positive	Positive
AD-11	Positive	Positive	Positive	Positive
AD-12	Positive	Positive	Positive	Positive
AD-13	Positive	Positive	Positive	Positive
AD-14	N/A	Positive	N/A	Positive
AD-15	Positive	Positive	Positive	Positive
AD-16	Positive	Positive	Positive	Positive
LBD-1	Positive	Positive	Positive	Negative
LBD-2	Positive	Positive	Positive	Positive
LBD-3	Positive	Positive	Positive	Positive
LBD-4	Positive	Positive	Positive	Positive
LBD-5	Positive	Positive	Positive	Positive
LBD-6	Positive	Positive	Positive	Positive
LBD-7	Positive	Positive	Positive	Positive
LBD-8	Positive	N/A	Positive	N/A
LBD-9	Positive	N/A	Positive	N/A
PART-1	Positive	Negative	Positive	Positive
PART-2	Positive	Negative	Positive	Negative
PART-3	Negative	Negative	Positive	Negative
PART-4	Positive	Negative	Positive	Positive
PART-5	Positive	Negative	Negative	Negative
PART-6	Positive	Positive	Positive	Positive
Control-1	Negative	Positive	Positive	Negative
Control-2	Negative	Negative	Negative	Negative
Control-3	Positive	Negative	Positive	Negative
Control-4	Positive	Negative	Negative	Negative
Control-5	Positive	Positive	Positive	Positive
Control-6	Positive	N/A	Positive	N/A
Control-7	Positive	Positive	Positive	Negative
Control-8	Positive	Negative	Positive	Negative

Supplemental Table 4. Case-by-case manual categorization based on the presence (positive) or absence (negative) of Tau pathology. N/A indicates a region of interest is not available.
